# The Diploma of the College of Surgeons Obtained under False Representations

**Published:** 1847-07

**Authors:** 


*The Diploma of the College of Surgeons obtained under false representations.*—The Taunton and Somerset Branch of the Provincial Medical and Surgical Association, having discovered that a Mr. James Dore Blake had obtained the diploma of the College of Surgeons by improper means, brought the matter before the Council. It appears that Mr. Blake had been for thirteen years prior to May, 1845, a retail pastrycook, and that after one year of Medical study he presented himself at the College of Surgeons, London, for examination, and obtained their letters testimonial as to his fitness to practise. The council, after considerable delay, sent the following resolution to Dr. Woodforde, the president of the branch association:—"At an extraordinary meeting of the council of the Royal College of Surgeons of England, Tuesday, the 27th of April, resolved, 'That it appears to the council that Mr. James Dore Blake obtained his examination and letters testimonial by false statements and imposition; and the council does, therefore, recal such letters testimonial, and hereby declares the same to be void; also, that Mr. Blake be requested to return the diploma granted to him, he having ceased to be a member of this college.'—EDMUND BELFOUR, Sec."—*London Med. Times.*

